# Differential susceptibility between skin and vaginal mucosa in sensitization phase of allergic contact dermatitis in mice

**DOI:** 10.1002/iid3.351

**Published:** 2020-09-11

**Authors:** Taku Nishijo, Kanako Nakayama, Masaaki Miyazawa, Yasutaka Kuroda, Hitoshi Sakaguchi

**Affiliations:** ^1^ Safety Science Research Laboratories Kao Corporation, Ichikai, Haga Tochigi Japan

**Keywords:** allergic contact dermatitis, contact hypersensitivity, draining lymph node cell, skin, vaginal mucosa

## Abstract

**Introduction:**

Mechanisms underlying skin sensitization in allergic contact dermatitis have been actively studied using the murine contact hypersensitivity (CHS) model. However, much less is known about sensitization at the vaginal mucosa (VM).

**Methods:**

We developed a CHS model with VM sensitization and epicutaneous elicitation at the ear. We then examined the proliferation activity of lymphocytes, the frequencies of T cells and the differentiation of hapten‐specific T cells in draining lymph nodes (dLNs) after sensitization.

**Results:**

Hapten‐specific CHS responses to 2,4‐dinitrofluorobenzene (DNFB), 2,4,6‐trinitrochrolobenzene, and oxazolone assessed by ear swelling suggested that the VM would be an inductive site of CHS to haptens. In the comparisons of CHS responses to each of the three haptens examined, the lower responses in VM‐sensitized mice were observed than skin‐sensitized mice (e.g., DNFB‐induced responses, −56%; *p* < .001, at 48 h after challenge). Consistent with the CHS responses, the DNFB‐induced proliferation of cells in dLNs examined by 5‐bromo‐2ʹ‐deoxyuridine assay was lower (−62%; *p* < .001) in VM‐sensitized mice than skin‐sensitized mice. On the other hand, between skin and VM sensitization, no significant differences were observed in the frequencies of interferon‐γ‐producing CD4^+^ and CD8^+^ effector, and regulatory T cells in dLNs after sensitization. We also observed no significant differences with respect to differentiation of hapten‐specific T cells based on the examination of cytokine production from dLN cells stimulated in vitro with 2,4‐dinitrobenzene sulfonate.

**Conclusion:**

These findings suggested that the lower T cell proliferation after VM sensitization is important for the lower CHS responses with VM sensitization than skin sensitization.

## Graphical abstract

We developed the murine contact hypersensitivity model that has explored with vaginal mucosal sensitization and epicutaneous elicitation at the ear. In the comparison of contact hypersensitivity responses to haptens, the lower ear swellings in vaginal mucosal sensitized mice were observed than that in skin sensitized mice. The data suggested that the induced proliferation of draining lymph node cells, but not the frequency of T cells in draining lymph nodes, may be a critical factor contributing to the lower contact hypersensitivity responses in vaginal mucosal sensitized mice.

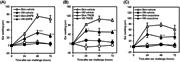

## 1 INTRODUCTION

Allergic contact dermatitis (ACD) is a skin inflammation that results from contact to specific allergens, that is, low‐molecular‐weight chemicals called haptens. The murine contact hypersensitivity (CHS) is one of the most frequently used animal models of ACD, characterized by the initial sensitization and subsequent elicitation phase.^1^ In the sensitization phase, antigen‐presenting cells (APCs) such as dendritic cells (DCs) ingest and process the hapten‐carrier complex, and then migrate to draining lymph nodes (dLNs) and present the antigen to naive T cells. This results in the generation of interferon‐γ (IFN‐γ)‐secreting CD4^+^ and CD8^+^ T cells which have been classically considered as the effector cells.^2^, ^3^ In the elicitation phase, a previously presented sensitizer triggers an infiltration of immune cells, such as antigen‐specific (effector) memory T cells.^4^, ^5^, ^6^ T cells release pro‐inflammatory cytokines and further stimulate cytotoxic CD8^+^ effector T cells and innate immune cells. This results in the development of a type IV hypersensitivity reaction.^7^ Previous studies have also demonstrated a central role for regulatory T (Treg) cells in the control of CHS due to its capacity to suppress CD8^+^ effector T cells in both the sensitization^8^, ^9^ and the elicitation phase.^10^, ^11^

Not only skin but also mucosal surfaces are exposed to a diversity of allergens. Type II mucosal surfaces such as the vaginal and oral mucosae (VM and OM, respectively) share many features in common with the skin, and are remarkably different from type I mucosae such as the intestinal and respiratory mucosae.^12^ For example, surfaces of type I mucosae are covered with a monolayer of epithelial cells and contain mucosa‐associated lymphoid tissue (MALT). In contrast, type II mucosae are lined with stratified squamous epithelia, and MALT is not found in steady state. Similar to the skin, Langerhans cells (LCs) and submucosal DCs (the counterpart of dermal DCs in the skin) reside within and beneath type II mucosal epithelial layers, respectively.

At the vulvar area, contact allergy can also be caused by exposure to contact allergens such as fragrance, preservative, and medicament.^13^, ^14^ The vulvar epithelium has more complex anatomical features than does the skin. The cutaneous epithelium of the mons pubis and labia majora, like the skin, is derived from the embryonic ectoderm and exhibits a stratified and keratinized structure. The degree of keratinization decreases over the clitoris and the outer surface and inner two‐thirds of the labia minora; the vulvar epithelium is not keratinized from the inner third of the labia minora through the vulvar vestibule. The VM is endodermal and mesodermal embryonic origin, a derivation shared with the lower portion of the vaginal tract.^15^ The non‐keratinized vulvovaginal mucosae morphologically resemble the non‐keratinized OM. These sites have 10‐ to 20‐fold higher permeability than the keratinized skin due to several factors such as the absence of a principal barrier.^16^, ^17^ As such, the exposure to contact allergens at the VM may present higher risk of contact allergy than that of the skin.

Contact allergy at the OM of type II mucosa, although seen less frequently, is often described as stomatitis.^18^, ^19^ Previous studies using a murine CHS model suggested that the OM has the capacity of CHS with local and remote T cell‐mediated reaction like the skin, although the CHS response is lower in OM‐sensitized mice than in skin‐sensitized mice.^20^, ^21^, ^22^ However, in contrast to the OM, few studies have reported the details for sensitization at the VM.

Here, this study aimed to develop a murine CHS model using VM sensitization and to compare the susceptibility to sensitization at either the skin or the VM, by examining the CHS response after epicutaneous elicitation at the ear. We then examined the proliferation activity of lymphocytes, the frequencies of T cells and the differentiation of hapten‐specific T cells in dLNs in the induction phase.

## 2 MATERIALS AND METHODS

### 2.1 Animals

C57BL/6J female mice were purchased from Japan SLC and used at 6–8 weeks of age under specific pathogen‐free conditions. All experimental procedures were approved by the Kao Corporation Animal Care Committee, and all experiments followed the guidelines of the committee.

### 2.2 Haptens

2,4‐Dinitrofluorobenzene (DNFB), 2,4,6‐trinitrochrolobenzene (TNCB), oxazolone (4‐ethoxy‐methylene‐2‐phenyloxazol‐5‐1), and 2,4‐dinitrobenzene sulfonate (DNBS) were purchased from Sigma‐Aldrich.

### 2.3 CHS model

For skin sensitization, on three consecutive days (Day 0–2), the shaved abdominal (1 cm × 1 cm) skin of mice was applied 10 μl of 0.3% (v/v) DNFB, 3.0% (v/v) TNCB or 2.0% (v/v) oxazolone in olive oil. For VM sensitization, on Day 0–2, 10 μl of same hapten solutions were applied into the vaginal lumen (approximately 0.7 cm^2^). Olive oil was used as the vehicle so as not to produce an irritating effect at the VM which has non‐keratinized epithelium. On Day 5, the mice were challenged with 20 μl of 0.3% (v/v) DNFB, 0.5% (v/v) TNCB or 0.5% (v/v) oxazolone in acetone/olive oil (4:1) (AOO), which were applied onto the dorsal and ventral surface of right ear. The left ear was painted with 20 μl of vehicle (AOO). Ear thickness was measured before challenge and at 24, 48, and 72 h after challenge at a predetermined site with a digital thickness gauge (Mitsutoyo). Ear swelling was calculated as follows: (right ear thickness after minus before challenge) − (left ear thickness after minus before challenge).

### 2.4 5‐Bromo‐2’‐deoxyuridine (BrdU) assay

On Day 4, mice were intraperitoneally injected with BrdU, which is an analogue of thymidine that incorporates into the DNA of proliferating cells. Mice were sacrificed on Day 5. Single‐cell suspensions were prepared from dLNs. As dLNs, we collected the axillary and inguinal LNs from mice with abdominal skin sensitization, and the inguinal and iliac LNs from mice with VM sensitization.^12^ BrdU‐ and 7‐aminoactinomycin (7‐AAD)‐positive cells (i.e., cells in the S phase of the cell cycle) were stained using BrdU assay kit purchased from BD Biosciences according to the manufacturer's instructions and counted with flow cytometry using the FACSVerse flow cytometric system (BD Biosciences). The results were analyzed with FlowJo software (FlowJo LLC). The number of BrdU^+^7‐AAD^+^ lymph node cells (LNCs) was calculated as follows: number of BrdU^+^7‐AAD^+^ cells = % of BrdU^+^7‐AAD^+^ cells × number of LNCs.

### 2.5 Antibodies and flow cytometry

Anti‐CD3ε (145‐2C11), anti‐CD4 (RM4‐5), anti‐CD8α (53‐6.7), anti‐CD25 (PC61), anti‐FoxP3 (FM23), anti‐inducible co‐stimulator (ICOS) (CD278; E7.17G9), and anti‐IFN‐γ (XMG1.2) antibodies and matching isotype controls were all purchased from BD Biosciences. Isolated LNCs were preincubated with anti‐CD16/32 (2.4G2; BD Biosciences) to block FcγR and were subjected to multicolor staining of cell surface; intracellular staining was facilitated by BD Cytofix/cytoperm fixation/permeabilization solution kit (BD Biosciences). Intracellular IFN‐γ staining was performed using leukocyte activation cocktail, with the BD GolgiPlug™ kit (BD Biosciences) including phorbol 12‐myristate 13‐acetate (PMA), a calcium ionophore (ionomycin) and the protein transport inhibitor BD GolgiPlug™ according to the manufacturer's instructions.

LNCs stained with antibodies were analyzed by flow cytometry using the FACSCanto II flow cytometric system or the FACSVerse flow cytometric system (both instruments were from BD Biosciences), and the results were analyzed with FlowJo software.

### 2.6 Cell culture and enzyme‐linked immunosorbent assay

Complete culture medium consisting of RPMI 1640 (Invitrogen) containing 10% heat‐inactivated fetal calf serum, 50 µM 2‐mercaptoethanol, 2 mM l‐glutamine, 25 mM *N*‐2‐hydroxyethylpiperazine‐*N*‐9‐2‐ethanesulfonic acid, 1 mmol/L nonessential amino acids, 1 mmol/L sodium pyruvate, 100 units/ml penicillin, and 100 μg/ml streptomycin was used. For DNBS‐dependent proliferation, single‐cell suspensions were prepared from LNs of mice on Day 5 of the treatment protocol. One million LNCs were cultured with 200 μg/ml DNBS for 72 h. The levels of IFN‐γ, interleukin‐4 (IL‐4), and IL‐17 in culture supernatants were measured by 
enzyme‐linked immunosorbent assay (ELISA) kits from R&D Systems (Minneapolis) according to the manufacturer's instructions.

### 2.7 Statistical analysis

Differences between two groups were analyzed by the two‐tailed Student *t* test, and it was considered statistically significant when *p* values were <.05.

## 3 RESULTS

### 3.1 Comparison of CHS responses among skin‐ and VM‐sensitized mice

**Figure 1:**
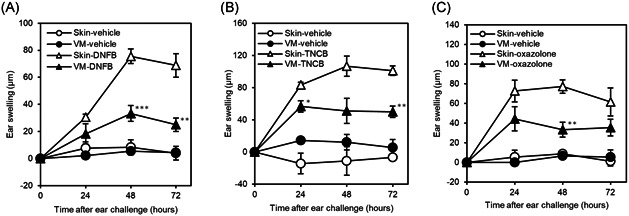
CHS responses at indicated times after challenge in skin‐ and VM‐sensitized mice. (a) DNFB‐induced responses. Data represent mean ± *SEM* (*n* = 6) and are representative of three independent experiments with similar results. (b) TNCB‐induced responses. Data represent mean ± *SEM* (*n* = 3). (c) Oxazolone‐induced responses. Data represent mean ± *SEM* (*n* = 3–6) and are representative of two independent experiments with similar results. CHS, contact hypersensitivity; DNFB, 2,4‐dinitrofluorobenzene; TNCB, 2,4,6‐trinitrochrolobenzene; VM, vaginal mucosa. *p < .05; **p < .01; ***p < .001, compared to skin‐sensitized mice

We compared CHS responses to the haptens of DNFB, TNCB, and oxazolone by either skin and VM sensitization, at the inductive and challenge dose optimized for maximal CHS response without irritant response. While the DNFB‐specific CHS responses peaked at 48 h after the challenge was developed in both skin‐ and VM‐sensitized mice, vehicle‐control mice with either skin or VM application did not develop skin inflammation (Figure 1A). Compared to skin‐sensitized mice, ear swelling was significantly lower in VM‐sensitized mice, with reductions of 56% and 64% at 48 and 72 h after challenge, respectively (Figure 1A).

This lower hapten‐specific CHS response in VM‐sensitized mice was also observed for other haptens of TNCB and oxazolone (Figure 1B,C). The TNCB‐specific responses of VM‐sensitized mice were significantly lower compared to that of skin‐sensitized mice, with reductions of 32% and 51% at 24 and 72 h, respectively (Figure 1B). Likewise, the oxazolone‐specific response of VM‐sensitized mice was significantly 57% lower at 48 h (Figure 1C).

Of note, we confirmed the area of the vaginal lumen which was exposed to the haptens was smaller than that of the skin, as described in Section Methods, 2. Thus, the exposed amount of hapten per unit area during sensitization was considered not to be smaller in the VM than in the skin. These data indicate that the VM is an inductive site of CHS to haptens, and the CHS response in VM‐sensitized mice is lower than that in skin‐sensitized mice.

### 3.2 Cell proliferation in dLN after hapten sensitization

**Figure 2:**
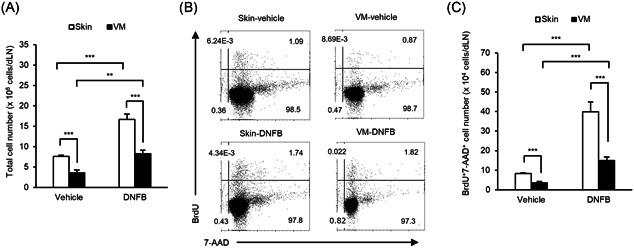
Induced dLNC proliferation by DNFB sensitization in skin‐ and VM‐sensitized mice. BrdU was injected intraperitoneally on Day 4 and dLNCs were collected on Day 5; cell proliferation was evaluated by flow cytometry. Total dLNCs (a), representative dot plots of LNCs (b) and the number of BrdU^+^7‐AAD^+^ cells (c) are as shown. Numbers associated with the dot plots (b) indicate percent cells in each quadrant. Data represent mean + *SEM* (*n* = 6) and are representative of two independent experiments with similar results. 7‐AAD, 7‐aminoactionomycin; BrdU, 5‐bromo‐2'‐deoxyuridine; DNFB, 2,4‐dinitrofluorobenzene; LNC, lymph node cell; VM, vaginal mucosa. **p < .01; ***p < .001

We next evaluated induced proliferation of lymphocytes in dLNs (the abdominal skin draining axillary/inguinal LNs or the vaginal draining inguinal/iliac LNs) after hapten application. The number of total draining lymph node cells (dLNCs) was significantly higher in DNFB‐sensitized mice than in vehicle‐control mice, with both skin and VM sensitization (Figure 2A). In addition, the number of total dLNCs was significantly lower in VM‐control mice than in skin‐control mice, and in VM‐sensitized mice than in skin‐sensitized mice (Figure 2A).

The frequency of BrdU^+^7‐AAD^+^ cells in dLNs was higher in DNFB‐sensitized mice than in vehicle‐control mice, with both skin and VM sensitization (Figure 2B). The frequencies of BrdU^+^7‐AAD^+^ cells were comparable between skin‐ and VM‐sensitized mice (Figure 2B). Furthermore, the number of BrdU^+^7‐AAD^+^ LNCs was significantly higher in DNFB‐sensitized mice than in vehicle‐control mice, with both skin and VM sensitization (Figure 2C). Proliferation in skin‐sensitized mice was 4.8‐fold higher compared to that in vehicle‐control mice. In addition, proliferation in VM‐sensitized mice was 4.3‐fold higher compared to that in VM‐control mice, which is similar to that of skin sensitization. As anticipated, the number of BrdU^+^7‐AAD^+^ cells was 62% lower in VM‐sensitized mice when compared to skin‐sensitized mice (Figure 2C). These findings are consistent with CHS responses.

### 3.3 T cell frequencies in dLN after hapten sensitization

**Figure 3:**
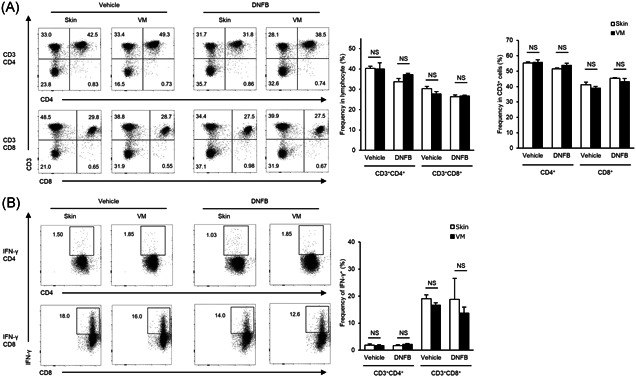
Flow cytometric analysis of CD4^+^ and CD8^+^ effector T cells in dLNs after DNFB sensization, collected from skin‐ and VM‐sensitized mice on Day 5. (a) Representative dot plots (left) and the mean frequencies of CD3^+^CD4^+^ and CD3^+^CD8^+^ T cells (middle) within the lymphocyte gate, and the mean frequencies of CD4^+^ and CD8^+^ T cells within the CD3^+^ gate (right). (b) Representative dot plots (left) and the mean frequencies of IFN‐γ‐producing cells (right) within gated CD3^+^CD4^+^ and CD3^+^CD8^+^ T cells. Numbers associated with the dot plots indicate percent cells in each gate. Data represent mean + *SEM* of at least *n* = 3 mice per group and are representative of two independent experiments with similar results. DNFB, 2,4‐dinitrofluorobenzene; LNC, lymph node cell; NS, not significant; VM, vaginal mucosa

To evaluate whether the differences in the frequencies of effector T cells in dLN after hapten sensitization would affect the distinct CHS responses at the ear, upon ex vivo restimulation with PMA and ionomycin, dLNCs collected from mice on Day 5 after DNFB sensitization were analyzed. Flow cytometric analysis showed no significant differences in the frequency of CD3^+^CD4^+^ T cells in the lymphocytes between skin‐control and VM‐control mice, and between skin‐ and VM‐sensitized mice (Figure 3A). Similar results were obtained for CD3^+^CD8^+^ T cells (Figure 3A). There were also no significant differences in the frequencies of CD4^+^ and CD8^+^ cells in CD3^+^ T cells between skin‐control and VM‐control mice, and between skin‐ and VM‐sensitized mice (Figure 3A). We then examined the frequencies of IFN‐γ‐producing cells in CD3^+^CD4^+^ and CD3^+^CD8^+^ T cells. Flow cytometric analysis with intracellular cytokine staining revealed no significant differences in the frequencies of IFN‐γ‐producing cells in either CD3^+^CD4^+^ or CD3^+^CD8^+^ T cells between skin‐treated and VM‐treated mice (Figure 3B).

**Figure 4:**
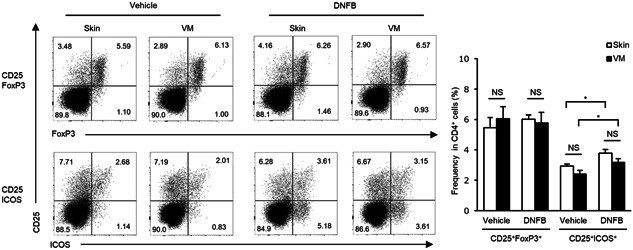
Flow cytometric analysis of Treg cells in dLNs after DNFB sensitization, collected from skin‐ and VM‐sensitized mice on Day 5. Representative dot plots (left) and the mean frequencies of CD25^+^FoxP3^+^ and CD25^+^ICOS^+^Treg cells (right) within the CD4^+^ gate are as shown. Numbers associated with the dot plots indicate percent cells in each quadrant. Data represent mean + *SEM* (*n* = 5–6) and are representative of two independent experiments with similar results. dLN, draining lymph node; DNFB, 2,4‐dinitrofluorobenzene; FoxP3, forkhead box P3; ICOS, inducible co‐stimulator; NS, not significant; VM, vaginal mucosa. *p < .05

In addition, we evaluated the frequency of Treg cells in CD4^+^ cells in response to DNFB sensitization. This analysis revealed that there were no significant differences in the frequencies of CD25^+^FoxP3^+^ Treg cells in CD4^+^ cells between skin‐control and VM‐control, and between skin‐sensitized and VM‐sensitized mice (Figure 4). Furthermore, the frequency of CD25^+^ICOS^+ ^Treg cells, which has been defined as a highly suppressive Treg cell population in CHS,^9^ in CD4^+^ cells was evaluated. DNFB‐sensitized mice showed a significantly higher frequency of CD25^+^ICOS^+ ^Treg cells than vehicle‐control mice in both skin and VM sensitization, while no significant differences were observed in skin‐treated and VM‐treated mice (Figure 4). Taken together, these data provide no evidence for the differential frequencies of effector T or Treg cells in dLNs, in a comparison between skin‐ and VM‐sensitization.

### 3.4 Cytokine production by hapten‐specific T cells in dLNs

**Figure 5:**
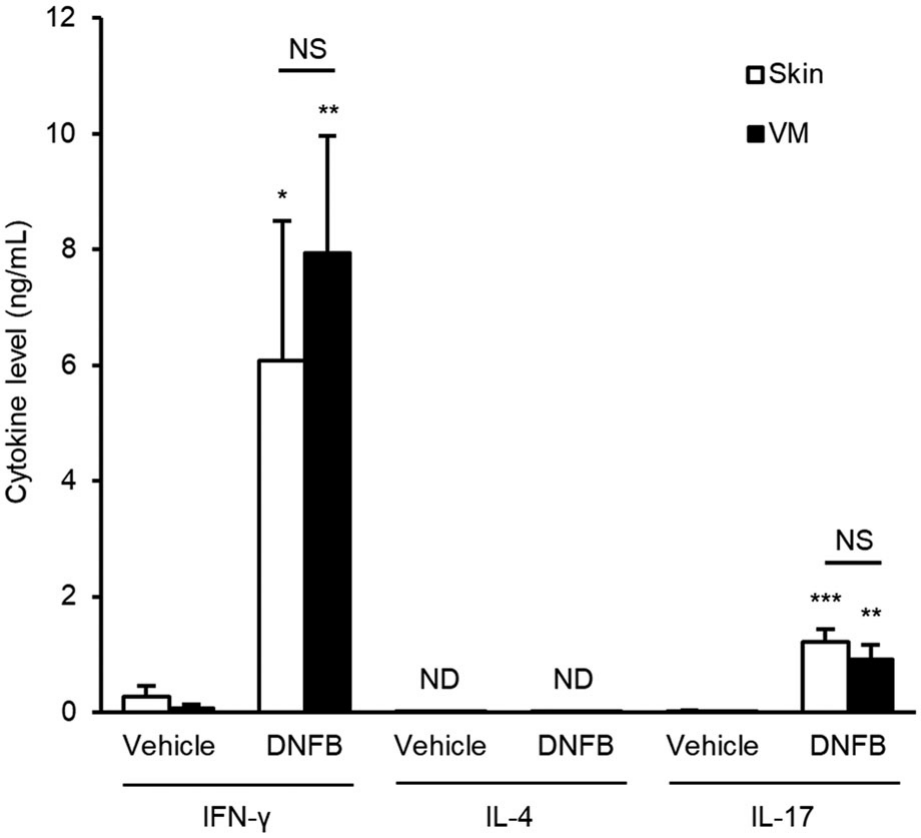
Levels of IFN‐γ, IL‐4, and IL‐17 detected in supernatants from dLNC cultures from skin‐ and VM‐sensitized mice. Cells were cultured ex vivo for 72 h with 200 μg/ml DNBS. Data represent mean + *SEM* (*n* = 5–6) and are representative of two independent experiments with similar results. dLN, draining lymph node; DNBS, 2,4‐dinitrobenzene sulfonate; DNFB, 2,4‐dinitrofluorobenzene; FoxP3, forkhead box P3; ICOS, inducible co‐stimulator; NS, not significant; VM, vaginal mucosa. *p < .05; **p < .01; ***p < .001, compared to vehicle‐control

To evaluate the differentiation of hapten‐specific T cells, the same number of one million dLNCs collected from each treatment group on Day 5 of the treatment protocol were stimulated in vitro with DNBS, which is a water‐soluble analogue of DNFB. Cytokines released into the culture supernatants were evaluated by ELISA 72 h after stimulation. The analysis revealed that the levels of IFN‐γ and IL‐17 induced by the DNBS stimulation in vitro were significantly higher in dLNCs from DNFB‐sensitized mice than in those from vehicle‐control mice in both skin‐ and VM‐sensitization, while no significant differences were observed between the responses of dLNCs from skin‐ and VM‐sensitized mice (Figure 5). IL‐4 was not detectable under any conditions. We also examined the differentiation of hapten‐specific T cells in several non‐dLNCs; these included the popliteal/axillary LNs of VM‐sensitized mice and the popliteal/iliac LNs of skin‐sensitized mice. No production of IFN‐γ was detected in cultures of non‐dLNCs stimulated with DNBS (data not shown). These findings reveal that there was no significant difference in the differentiation of hapten‐specific T cells in dLNs after sensitization between skin‐ and VM‐sensitization; these findings are consistent with the T cell frequencies observed in dLNs.

## 4 DISCUSSION

In this study, we used a murine model of CHS to compare the allergic responses to skin and VM sensitization. This is, to our knowledge, the first murine CHS model that has explored hapten sensitization via the VM. The data suggested that the VM is an inductive site of CHS responses to haptens. In the comparison of CHS responses to DNFB, TNCB, or oxazolone, the lower ear swellings in VM‐sensitized mice were observed than that in skin‐sensitized mice. Consistent with these responses, cell proliferation in dLNs was lower in VM‐sensitized mice than skin‐sensitized mice. On the other hand, between skin and VM sensitization, we detected no significant differences in the frequencies of effector T and Treg cells in dLNs after DNFB sensitization, and the differentiation of hapten‐specific T cells in dLNs. These findings suggested that the induced proliferation of dLNCs, but not the frequency of T cells in dLNs, may be a critical factor contributing to the lower CHS responses in VM‐sensitized mice.

One possible explanation for the lower CHS response in VM‐sensitized mice may be that there are distinct subsets of APCs in the skin and VM. APCs in the skin of mice are categorized into some subsets which drive distinct but overlapping T cell phenotype.^23^ The epidermis is populated by LCs, and the dermis is populated by at least two subsets of dermal DCs (e.g., CD103^+^ DCs and CD11b^+^ DCs). Whereas a single homogenous population of CD207^+^ LCs has been found in the skin epidermis, at least three populations of APCs with very low expression of CD207 have been found in the vaginal epithelium of mice in steady state.^24^ It is also interesting to note that the DC subset as counterparts for CD103^+^ DCs, which are considered to be the central APCs for the induction of T‐helper (Th) 1/T‐cytotoxic 1 cells in CHS at the skin,^23^, ^25^ has not been reported for the vaginal submucosa.^26^ Collectively, the observed differential susceptibility to sensitization between the skin and VM could be accounted for in part by dissimilarities of APCs as described above.

Another explanation for the differential susceptibility may relate to the differences in steady state cell number within dLNs, the site at which APCs present antigen to naive T cells. Axillary/inguinal LNs from mice with abdominal skin sensitization and inguinal/iliac LNs from mice with VM sensitization were collected as dLNs in this study. Because the number of iliac LNCs was smaller than that of axillary LNCs, the total dLNC number was smaller in the VM than in the skin. Nonetheless, the proliferation rate of dLNCs from VM‐sensitized mice was similar to that observed for skin‐sensitized mice (Figure 2A–C). We confirmed that the differentiation of hapten‐specific T cells after sensitization was not shown in non‐dLN (i.e., the popliteal/axillary LNs of VM‐sensitized mice and the popliteal/iliac LNs of skin‐sensitized mice). Thus, the number of dLNCs identified before sensitization may also be a contributing factor to the different susceptibilities to sensitization.

Furthermore, the epithelial surfaces of VM are covered with mucus, which protects against viral entry and contains secretory proteins with microbicidal and antiviral activity. Mucus may dilute, buffer, or neutralize haptens. In addition, as has been considered with respect to the OM, the specific anatomic structure may help rapid dispersion and absorption of haptens, consequently prevent from prolonged contact of the hapten.^19^ This may also be one reason for the lower CHS response in VM sensitized mice considering its morphological similarity to the OM.

Our study featured haptens that typically induce Th1 responses (including production of IFN‐γ). However, recent reports from human ACD studies have shown distinct immune polarization according to allergen.^27^ Although a previous study indicated that oxazolone may partly induce responses via different pathways of IL‐4 from DNFB and TNCB which have dinitrophenyl group,^28^ further investigations using a larger variety of haptens would be needed to improve the commonality of the findings in this study.

In conclusion, while the VM is an inductive site of CHS responses to haptens, the lower CHS responses in VM‐sensitized mice were observed than that in skin‐sensitized mice. The findings in this study suggested that the quantitative differences focused on the number of dLNCs, but not the frequency of T cells in dLNs, may be important toward improving our understanding of the lower CHS responses in VM‐sensitized mice. To address the reason for the differential susceptibilities to sensitization, it will be of interest to focus on the dissimilarities of APCs between skin and VM. It should also be considered that there are other conceivable factors which may contribute either alone and in combination, such as dLNC number in steady state and mucus, as described above. Future studies will address these issues.

## CONFLICT OF INTERESTS

The authors declare that there are no conflict of interests.

## AUTHOR CONTRIBUTIONS

*Study conception*: Taku Nishijo, Kanako Nakayama, and Masaaki Miyazawa. *Experimental design*: Taku Nishijo, Kanako Nakayama, Yasutaka Kuroda, and Masaaki Miyazawa. *Acquisition and analysis of data*: Taku Nishijo and Kanako Nakayama. *Interpretation of data*: all authors. *Writing*: Taku Nishijo.
